# Anemoside B4 mitigates endoplasmic reticulum stress-induced apoptosis post cerebral ischemia/reperfusion injury in rats

**DOI:** 10.22038/ijbms.2025.85441.18475

**Published:** 2025

**Authors:** Xiaohuan Huang, Ran Deng, Chaoyue Ma, Huizhi Fei

**Affiliations:** 1 Department of Pathology, Chongqing Three Gorges Medical College, Wanzhou, China; 2 Chongqing Three Gorges Medical College, Wanzhou, China; 3 Medical college, Jiujiang University, Jiujiang, Jiangxi Province , China

**Keywords:** Anemoside B4, Apoptosis, Cerebral ischemia/ - reperfusion injury, Endoplasmic reticulum – stress, MCAO/R

## Abstract

**Objective(s):**

Anemoside B4 (AB4) exhibits neuroprotective effects on cerebral ischemia/reperfusion injury (CIRI), and endoplasmic reticulum stress (ERS) plays a crucial role in the process of CIRI. Nevertheless, it remains unknown whether AB4 acts on CIRI via ERS. This study is designed to determine whether AB4 mitigates CIRI by suppressing ERS-induced neuronal apoptosis.

**Materials and Methods:**

One hundred thirty-five male SD rats (260–290 g) were randomly assigned to five groups, with 20 rats in each group: 1. Sham. 2. Middle Cerebral Artery Occlusion (MCAO/R). 3. AB4-L: Prior to MCAO, rats were subjected to continuous intraperitoneal injection of 1.25 mg/kg of AB4. 4. AB4-M: Rats were administered a continuous intraperitoneal injection of 2.5 mg/kg of AB4 prior to undergoing MCAO. 5. AB4-H: Rats were continuously intraperitoneally injected with 5 mg/kg of AB4 before MCAO. Additionally, another thirty-five rats were employed for the time point.

**Results:**

TTC staining results demonstrated that AB4 significantly reduced cerebral infarct volume. Histopathological analysis of brain tissues revealed that AB4 mitigated neuronal damage. In the MCAO model, GRP78 expression progressively increased with reperfusion time and peaked at 24 hr. AB4 treatment decreased mRNA levels of key ERS markers, including GRP78, ATF6, IRE1α, and PERK. Additionally, AB4 reduced protein levels of GRP78, p-PERK, PERK, ATF6, and p-IRE1α, further indicating its role in attenuating ERS. TUNEL results demonstrated that AB4 significantly reduced neuronal apoptosis.

**Conclusion:**

AB4 may serve as a potential therapeutic agent for CIRI, potentially exerting neuroprotective effects through inhibiting ERS-mediated apoptosis.

## Introduction

Stroke is characterized as an acute focal neurological deficit that can be exclusively attributed to a cerebrovascular etiology (1). Patients typically present with hemiparesis, aphasia, and sensory as well as visual impairments. Globally, acute arterial occlusion-induced ischemic strokes account for between 60% and 70% of all strokes and remain one of the leading contributors to mortality and disability worldwide. The most effective approach to treating ischemic stroke is the rapid restoration of blood flow; however, this re-establishment of circulation inevitably triggers a cascade of pathophysiological changes (2). In recent years, considerable attention has been focused on elucidating the pathophysiological mechanisms underlying ischemic stroke, including oxidative stress, apoptotic processes, neuroinflammation, and excitotoxicity (3). These processes are collectively termed ‘cerebral ischemia-reperfusion injury’ (CIRI), and comprehensive investigation is imperative to elucidate the underlying mechanisms.

When protein homeostasis is compromised and the endoplasmic reticulum (ER) accumulates misfolded or unfolded proteins, the ER stress (ERS) response is triggered (4). This response activates protein kinase RNA-like endoplasmic reticulum kinase (PERK), activating transcription factor 6 (ATF6) and inositol-requiring enzyme 1 (IRE1), thereby initiating an adaptive unfolded protein response (UPR) aimed at restoring proteostasis. Nevertheless, the URP plays a significant pathological role in various diseases, including metabolic disorders, cancer, and neurodegenerative conditions (5). Research has demonstrated that ERS is pivotal in CIRI (6). While moderate ERS can enhance cellular tolerance and facilitate the restoration of homeostasis, excessive or prolonged ERS may trigger the activation of apoptotic pathways (7). Following chronic and unresolved ERS, UPR can trigger apoptosis through various mechanisms involving glycogen synthase kinase 3/3β(GSK3/3β), c-Jun N-terminal kinase (JNK), CCAAT/enhancer-binding protein homologous protein (CHOP) or caspase-12. Apoptosis of neuronal cells results in their demise, thereby intensifying the neurological deficits (8). Targeting ERS may represent a promising therapeutic approach for CIRI.

Pulsatilla is a widely utilized traditional Chinese medicine. Its extracts and active constituents exhibit a diverse array of pharmacological effects, including anti-tumor, anti-inflammatory, antibacterial, and antiviral properties, as well as immune enhancement and anti-aging effects. It demonstrates significant anti-inflammatory, anti-apoptotic, anti-oxidative, enhancing autophagy effects. The bioactive compound present in pulsatilla, known as Anemoside B4 (AB4), has garnered considerable attention over the past two decades, resulting in numerous studies aimed at investigating its anti-inflammatory, immunoregulatory, and anti-tumor activities, including acute lung injury, renal damage, inflammatory pain, hepatocellular carcinoma, neurological diseases (9). Our previous investigations have demonstrated that AB4 exerts a protective effect against cerebral CIRI, with its pharmacological mechanisms encompassing anti-inflammatory and anti-apoptotic activities. Nevertheless, the study serves as a preliminary investigation into the effects of AB4, and the specific neuroprotective mechanisms require further elucidation.

In this study, we aimed to investigate the effects of AB4 on ERS and ERS-associated apoptosis in rats subjected to CIRI. Ultimately, the mechanisms underlying ERS-related apoptosis warrant further investigation.

## Materials and Methods

### Animals and experimental groups

A total of one hundred thirty-five male SD rats, 8–12 weeks, 260–290g, were provided by Hunan Slake Jingda Experimental Animal Co., Ltd. All animals were given *ad libitum* access to water and food and were housed in a temperature-controlled environment maintained at 24 ± 2 ℃ with humidity levels of 50–65%. They were kept under a 12-hour light/dark cycle. All rats were acclimated to feeding for one week prior to the experiment. The rats were promptly sacrificed after deep anesthesia without any pain.

**Figure 1:**
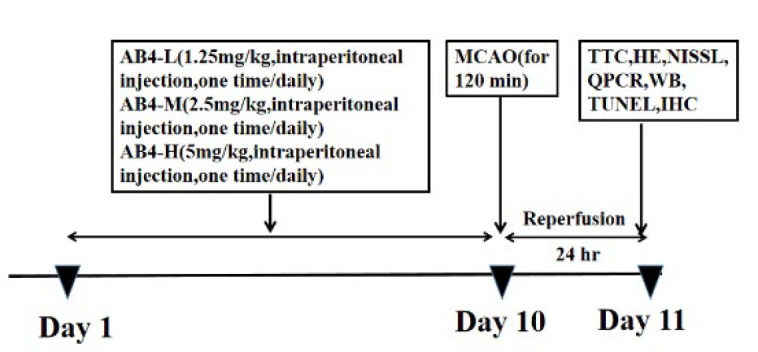
Schematic illustration of experiment protocol for MCAO/R

Animals were randomly classified into five groups: ①Sham group: The surgical procedures were identical to those in the model group, with the primary difference being that the left middle cerebral artery was not occluded. ② Middle Cerebral Artery Occlusion (MCAO/R) group: The left middle cerebral artery was occluded for 2 hr, followed by 24 hr of reperfusion. ③AB4-L group: The MCAO/R rat model was pre-administered with 1.25 mg/kg of AB4 once daily for ten consecutive days. ④AB4-M group: Before establishing the MCAO/R rat model, AB4 was administered once daily at a 2.5 mg/kg dose for ten consecutive days. ⑤AB4-H group: Before the rat model, AB4 was administered continuously at 5 mg/kg daily for 10 days. All drugs were dissolved in 0.9% normal saline and administered via intraperitoneal injection. The experimental timeline and design are illustrated in Figure 1.

### MCAO/R model

According to the steps described in the published literature (10, 11), MCAO surgery was performed on rats under anesthesia (3.5% chloral hydrate, 0.01 ml/g). The rats were placed supine, and an incision was made along the midline of the neck. After vascular separation, threading and knotting were performed. After the left internal carotid artery was clamped with an arterial clamp, a “V”-shaped incision was rapidly made on the left common carotid artery. The filament was inserted into the vessel along the incision, passing successively through the left common carotid and internal carotid arteries and ultimately reaching the middle cerebral artery (the black mark could be reached when passing through the bifurcation of the internal and external carotid arteries). The filament was gently pulled out two hours later, and the rats were treated 24 hr after reperfusion. The sham operation group had the same operation as the MCAO group, except no filament was inserted. 

### Neurological score

Twenty-four hours after reperfusion, the neurological function of all rats was evaluated based on the Longa five-point scale: 0, no neurological deficit; 1, inability to fully extend the contralateral forelimb; 2, circling to the contralateral side when walking; 3, falling to the contralateral side when walking; 4, inability to walk spontaneously or death. Only rat scores of 1, 2, or 3 were eligible for inclusion in the statistics and subsequent experiments (12, 13). 

### Cerebral infarction volume

Following the euthanasia of the rats, their heads were decapitated to extract the brains, which were immediately placed at -20 °C. After a period of 30 min, the brains were sectioned into slices at 2mm intervals (yielding a total of five slices) and subsequently immersed in preheated 2% 2,3,5-triphenyltetrazolium chloride hydrochloride (TTC) solution at 37 °C for 15 to 20 min. The brain slices were then inverted and subjected to the same staining procedure. Ultimately, the stained brain sections were transferred to a fixative solution containing 4% paraformaldehyde for two days. The brain slices were arranged sequentially from superior to inferior, photographed, and analyzed for infarction volume using ImageJ software. The formula utilized for calculating infarction volume is as follows: {[Total infarct volume - (Volume of the left cerebral hemisphere - Volume of the right cerebral hemisphere)] / Volume of the right cerebral hemisphere}× 100%. This calculation was conducted on five brain slices, and the average percentage of infarction foci was subsequently calculated (14, 15).

### Hematoxylin-eosin (HE) and Nissl staining

According to existing literature, the brain was harvested following 24 hr of reperfusion and promptly fixed in 4% paraformaldehyde. After an additional 24-hour fixation, brain tissue sections (2–3 mm thick) were extracted along the coronal axis from the hippocampal region. The samples underwent a series of processes, including dehydration, clearing, paraffin infiltration, embedding, sectioning, baking, and staining with hematoxylin and eosin (initially immersed in hematoxylin solution followed by differentiation until a blue hue was achieved before immersion in eosin). Nissl staining was performed by placing the sections in Nissl stain solution. Subsequent dehydration and clearing were conducted prior to sealing. Full-slide scanning was then performed for observation using computer analysis (16).

### Quantitative RT-PCR

Step 1: Total RNA was extracted from the cerebral cortex of rats using a total RNA extraction kit, and the concentration and purity of the RNA were evaluated for each group utilizing a spectrophotometer. Step 2: Based on the determined concentrations, the requisite amount of RNA for reverse transcription was calculated, followed by preparation of a 20-microliter reaction system with All-In-One 5X RT MasterMix reagent. The thermal cycling conditions were established: incubation at 37°C for 15min, reverse transcription at 60 ℃ for 10min, and termination of the reaction at 95 ℃ for 3min to yield cDNA. Step 3: A subsequent reaction system with a total volume of 20 microliters was prepared using BlasTaqTM 2X qPCR MasterMix kit. The enzyme was activated at 95 ℃ for 3min; denaturation occurred at this temperature for 15 sec, followed by annealing/extension at 60 ℃ for one minute. This process involved conducting 40 cycles alternating between denaturation and annealing/extension temperatures (95 ℃ and 60 ℃). β-actin served as an internal reference gene, with its relative expression level analyzed via the 2^−ΔΔCt^ method (17). Primer sequences are detailed in Table 1.

### Western blot

The rat cerebral cortex was extracted from a -80 ℃ freezer, weighed, and the required volume of freshly prepared RIPA lysis buffer (containing PMSF) was calculated. The tissue was homogenized evenly using a glass pestle, transferred to EP tubes, and subjected to sonication for 2 min. Subsequently, the sample was placed on ice for 30 min to facilitate lysis before being centrifuged at 4 °C and 12,000 rpm for 15 min to collect the supernatant while discarding the pellet. Protein concentrations in each group were determined using the BCA assay and standardized against the lowest concentration group. Following this, an SDS-loading buffer was added to the samples, which were then denatured in a metal bath at 99.5 ℃ for 5min. Electrophoresis was performed next, followed by transferring proteins onto PVDF membranes (pre-activated with methanol), which were incubated at room temperature with 5% skim milk for 1-2h to block non-specific binding sites. The primary antibodies of GRP78, *p*-PERK, PERK, *p*-IRE1a, IRE1a, and ATF6 were incubated overnight at 4 ℃. The corresponding secondary antibodies were subsequently incubated at room temperature for one hour or two based on primary antibody species before washing the membrane and performing ECL chemiluminescence to visualize protein bands. A semi-quantitative analysis of band gray values (the gray value of target bands relative to that of internal reference bands) was conducted using ImageJ software to calculate the relative expression levels of target proteins(18).

### TUNEL

The paraffin-embedded sections were dewaxed in water and gently dried, after which a histochemical pen was used to delineate a circle around the tissue to prevent fluid loss. Subsequently, a proteinase K working solution was added to the circle to cover the tissue and incubate at 37 ℃ for 20 min. Following three washes with PBS, a membrane-disrupting working solution was applied within the circle at room temperature for an additional 20 min. After washing, the sections were equilibrated at room temperature for 10min. Based on the number of slides and tissue size, appropriate amounts of TDT enzyme, dUTP, and buffer from the TUNEL kit were combined in a ratio of 1:5:50 before being added to cover the tissue within the circle. The slides were then placed flat in a humidified chamber and incubated at 37 ℃ for one hour. After washing, the DAPI staining solution was introduced into the circle and incubated under dark conditions at room temperature for 10 min. Finally, following slight drying of washed slides, they were mounted using an anti-fluorescence quenching agent before observation under a fluorescence microscope (16).

### Statistical

Data were processed using SPSS 25.0 software, and results are presented as mean ± SEM. Statistical significance was assessed through normality and lognormality tests(Shapiro-Wilk), followed by one-way analysis of variance for multiple comparisons. *P*-value<0.05 was considered indicative of statistical significance.

## Results

### Cerebral infarction volume and neurological deficit scores

**Figure 2:**
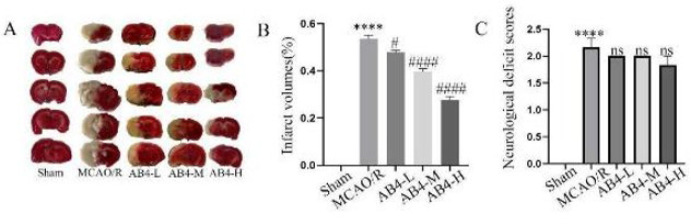
Effect of AB4 on cerebral infarct volume and neurological deficit score caused by CIRI in rats

Figure 2A presents the TTC staining images of cerebral infarction in different groups. Figure 2B displays the statistical data of infarction volume among different groups. The findings indicated a significant increase in both infarct volume and neurological score within the MCAO/R group (*P*<0.0001). In comparison to the MCAO/R group, both the medium and high dosage groups of AB4 demonstrated significant reductions in cerebral infarction volume(*P*<0.0001); the low dosage group showed a decrease, but it was not as significant as the previous two groups(*P*<0.05). Additionally, The neurological score across all three dosage groups did not exhibit significant differences when compared to the MCAO/R group(*P*<0.05) (Figure 2C).

### Brain tissue injury

**Figure 3:**
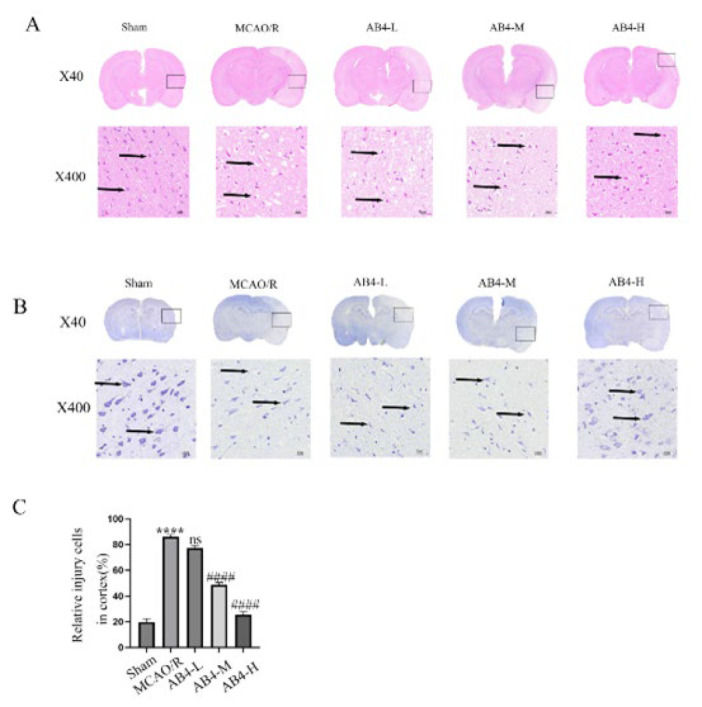
Effect of AB4 on brain tissue injury caused by CIRI

As illustrated in Figures 3A and 3C, the cortex of the MCAO/R group exhibited a loose and pale appearance, characterized by a significant reduction in neuronal density and numerous vacuoles surrounding the neurons; the number of damaged cells exhibited a significant increase. Treatment with AB4 significantly alleviated edema, reversed the decline in the number of neurons, and simultaneously significantly reduced vacuole formation and the number of damaged cells. Notably, high-dose AB4 demonstrated superior efficacy compared to both medium and low-dose treatments. As shown in Figure 3B, there was a significant reduction in the number of Nissl bodies within neurons of the MCAO/R group, with some Nissl bodies completely absent. Following AB4 intervention, the quantity of Nissl bodies in these neurons was restored. Notably, the high dose of AB4 exhibited a more pronounced effect than both medium and low doses. 

### Cerebral cortex GRP78 protein level 

**Figure 4:**
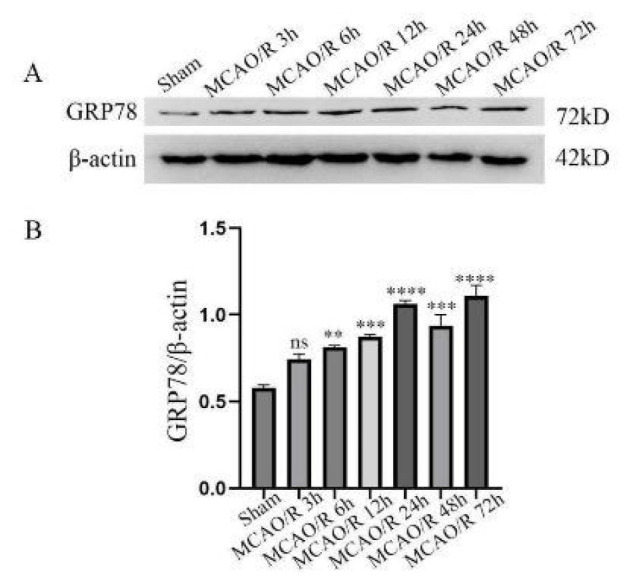
Expression levels of GRP78-induced MCAO/R in different reperfusion times

To investigate the changes in ERS after CIRI, we evaluated the expression levels of ERS-marked proteins in the cortical tissue of model animals at different time points after reperfusion. The study results showed a significant difference in the expression level of GRP78 protein at different time intervals after reperfusion (Figure 4A-B). After IR, the expression level of GRP78 exhibited a marked increase starting from 6 hr, peaked at 24 hr, and remained elevated at 48 hr and 72 hr. In summary, these findings suggest that GRP78 is activated in CIRI, implying ERS exists during the CIRI process.

### Cerebral cortex ERS related proteins mRNA level

**Figure 5:**
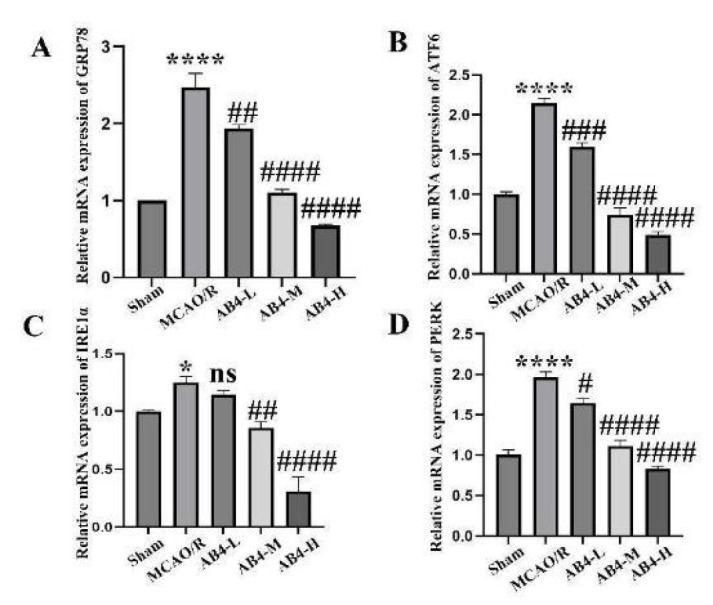
Effect of AB4 on mRNA expression levels of ERS-related proteins induced by MCAO/R. (A) The relative mRNA expression of GRP78. (B) The relative mRNA expression of ATF6. (C) The relative mRNA expression of IRE1α. (D) The relative mRNA expression of PERK. Data are presented as mean ± SEM (n=6). *P<0.05 and ****P<0.0001 vs Sham group; #*P*<0.05, ##*P*<0.01, ###*P*<0.001, and ####*P*<0.0001 vs MCAO/R group. MCAO/R, middle cerebral artery occlusion/reperfusion; AB4, Anemoside B4; AB4-L, low dosage of AB4 (1.25 mg/kg); AB4-M, middle dosage of AB4 (2.5 mg/kg); AB4-H, high dosage of AB4 (5 mg/kg); GRP78, glucose regulated protein 78kD;ATF6, activating transcription factor 6; IRE1α, inositol requiring enzyme 1α; PERK, protein kinase RNA-like endoplasmic reticulum kinase.

To assess the impact of AB4 intervention on the transcriptional levels of ERS-related proteins, we employed qRT-PCR to measure the mRNA expression levels of GRP78, ATF6, IRE1α, and PERK (Figure 5A-D). Compared with the Sham group, the mRNA levels of GRP78, ATF6, IRE1α, and PERK in the cortex of the MCAO/R group were significantly elevated (*P*<0.05 and *P*<0.0001); Compared with the MCAO/R group, the expressions of GRP78, ATF6, and PERK in the low dosage group were all reduced except for IRE1α (*P*<0.05 and *P*<0.001); in the medium and high dosage groups, the expressions of GRP78, ATF6, IRE1α, and PERK decreased gradually (*P*<0.01 and *P*<0.0001). The research findings demonstrate that AB4 significantly influences the mRNA levels of proteins associated with ERS during cerebral ischemia/reperfusion injury.

### Cerebral cortex ERS related protein level

**Figure 6:**
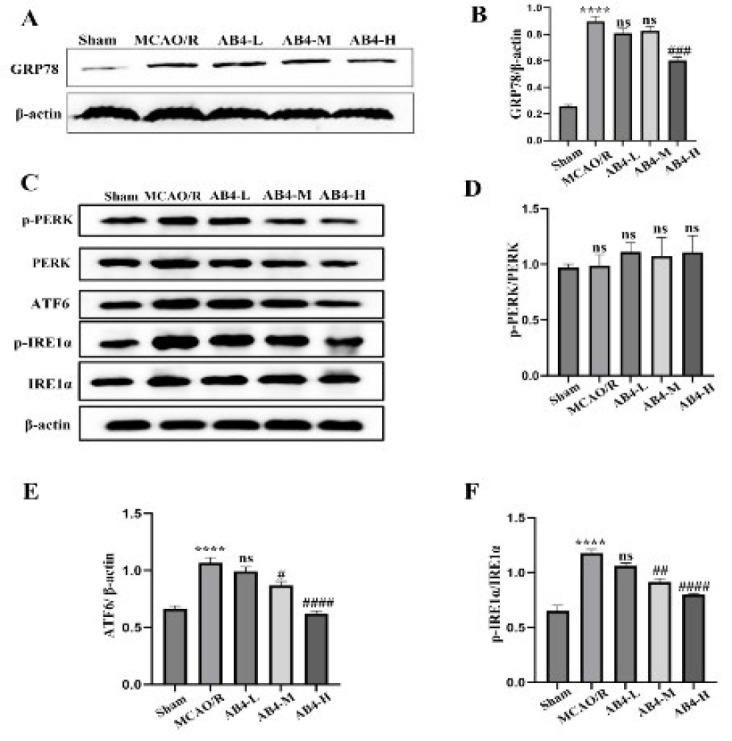
Effect of AB4 on expression levels of ERS-related proteins induced by MCAO/R

Figure 6 (A-F) exhibits the expression of ERS-related proteins in the cerebral cortex of all experimental groups (A, C) as well as the statistics (B, D-F). In contrast to the Sham group, GRP78, ATF6, and p-IRE1α protein levels were significantly elevated in the MCAO/R group (*P*<0.0001). Following AB4 treatment, the expression levels of GRP78, ATF6, and p-IRE1α were significantly reduced, with the most pronounced effect observed in the high dosage group (*P*<0.001 and *P*<0.0001). In contrast, no significant differences in the ratio of p-PERK/PERK were noted among the groups. In conclusion, AB4 significantly affects the expression levels of ERS-related proteins during cerebral ischemia/reperfusion injury.

### Cerebral cortex apoptosis

**Figure 7:**
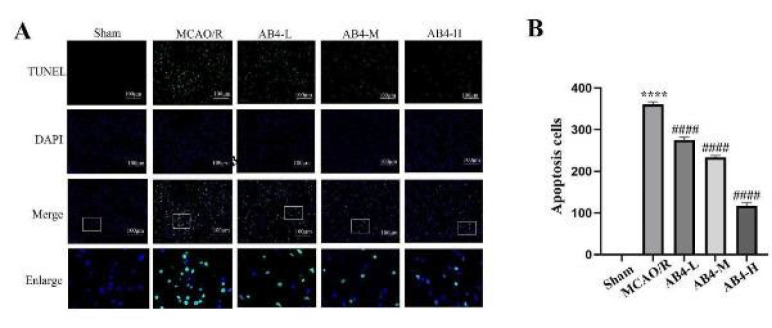
Effect of AB4 on apoptosis in the cortex following MCAO/R

To investigate the effect of AB4 on apoptosis in the CIRI model, TUNEL staining was used to detect apoptotic cells in the cerebral cortex (Figure 7A-B). In the Sham group, TUNEL-positive cells were scarcely detectable, whereas a considerable number of TUNEL-positive cells were identified in the MCAO/R group(*P*<0.0001). However, compared with the MCAO/R group, the AB4 treatment group exhibited a significantly lower number of TUNEL-positive cells (*P*<0.0001). These results confirm that AB4 can exert an anti-apoptotic effect during cerebral ischemia/reperfusion injury.

## Discussion

In this research, the MCAO/R model was employed to simulate the *in vivo* pathological process of CIRI(19), thereby facilitating the exploration of the neuroprotective effect of AB4. Our research outcomes demonstrated that the high-dose AB4 group could conspicuously reduce the volume of cerebral infarction and mitigate brain tissue damage and neuronal impairment. We also discovered that endoplasmic reticulum stress (ERS) was activated in CIRI, and AB4 could inhibit the activation of ERS-related proteins and apoptosis. Our previous *in vitro* studies have also verified that AB4 alleviated cell damage and apoptosis. These findings imply that AB4 might attenuate CIRI by suppressing ERS-mediated apoptosis. 

The endoplasmic reticulum (ER) is crucial in maintaining cellular homeostasis. ERS can trigger diverse cell death modalities by activating the UPR signaling pathway(20). Cell death plays a vital role in the occurrence and progression of diseases such as neurological disorders, cancer, and cardiovascular diseases(21). Studies have shown that esculin can trigger ER stress, thereby increasing the level of PERK protein and activating the PERK-eIF2α-CHOP and PERK-Nrf2-HO-1 signaling pathways(22); these pathways jointly promote apoptosis and ferroptosis of colorectal cancer cells *in vitro* and *in vivo*; ER stress-induced apoptosis in chronic cerebral hypoperfusion (CCH) is associated with the IRE1α/TRAF2/ASK1/JNK signaling pathway; Licochalcone A (LicA) can induce apoptosis in endometrial cancer (EMC) cells through the GRP78-mediated ER-stress pathway. In the present research, rats were first subjected to MCAO for two hours, followed by reperfusion at six distinct time points. The expression alterations of the ERS marker GRP78 were observed. The WB results demonstrated that ERS occurs during CIRI, whereas the TUNEL results suggested that AB4 can suppress cell apoptosis following CIRI. 

Organ ischemia is likely to result in irreversible tissue damage. Tissue reperfusion is employed to prevent further ischemia; nevertheless, in certain circumstances, it might aggravate the injury via a process referred to as ischemia-reperfusion (I/R) injury. The key pathological processes resulting in I/R injury encompass apoptosis, inflammatory response, oxidative stress, fibrosis, ferroptosis, and blood-brain barrier damage (23). Our research findings also suggest that following the MCAO/R model, the brain tissue injury in rats was significantly exacerbated, with a notable increase in cerebral infarction volume and neurological function scores; after AB4 treatment, the brain tissue injury could be obviously mitigated, and the infarction volume decreased; however, there was no significant change in the neurological function score. Researchers opine that this might be associated with the weight of the selected rats. The weight of the rats in this research was within the range of 260 – 290 g. During the MCAO/R procedure, the mortality rate of the rats was significantly reduced compared to the previous literature reports. The neurological function scores of the rats in the model group were almost all 2 points, with no rats scoring 3 or 4 points. It is probable that rats with larger body weight have better tolerance to this model, leading to an insignificant change in the neurological function scores after AB4 treatment.

CIRI results in the accumulation of unfolded or misfolded proteins in ER and triggers the ERS response. During ERS, GRP78 dissociates from transmembrane receptors: PERK, IRE1, and ATF6; then, these receptors are autophosphorylated and activate their signaling pathway to maintain normal physiological function, however, prolonged and excessive ERS lead to apoptosis and neuronal injury. ERS plays a key role in the pathophysiological processes of CIRI. Blocking neuronal apoptosis induced by ERS can protect neuronal function and alleviate CIRI(24). In this study, we found that protein and mRNA expression levels of GRP78, ATF6, and phosphorylated forms of PERK and IRE1α were significantly increased in the MCAO/R group, indicating the activation of ERS induced by CIRI. AB4 reversed the growing trend in a dose-dependent manner. The results indicated that ERS occurred induced by CIRI, and AB4 alleviated CIRI by suppressing ERS.

Interestingly, there were disagreements among the results of our study. Firstly, the neurological function scores of the model group were all 2 points, and so were those of the AB4 treatment group. Theoretically, AB4 is supposed to exert a neuroprotective effect, and thus the neurological function scores should have changed. However, such a change was not observed. Secondly, in our study, the ratio of *p*-PERK/PERK showed no significant changes among groups; however, the expression of *p*-PERK and PERK obviously changed, parallel to the mRNA level of PERK. This finding is different with previous studies, which showed the expression of PERK has no apparent alternation, but the ratio of *p*-PERK/PERK elevated in CIRI (15). Another report showed that the mRNA expression of PERK increased in CIRI, consistent with our results (25). Moreover, it (26) also has been reported that the protein expression of PERK and *p*-PERK increased in MCAO/R rats; at the same time, the ratio of *p*-PERK/PERK was markedly elevated, similar to our results. Our results and previous studies suggested that the activation of the PERK signal pathway might be not only by auto-phosphorylating to increase the phosphorylated form but also by increasing the expression level of PERK to increase the phosphorylated PERK. 

This study also presents several limitations. To begin with, the effect and mechanism of AB4 were explored only *in vivo*, not *in vitro*. Secondly, the relationship between ERS and apoptosis was not explored in depth. Only the TUNEL assay was performed, while the apoptotic pathway markers caspase-12 and caspase-3 were not detected. Finally, further investigation into the mechanism underlying the role of AB4 in ERS was not conducted. 

The *in vitro *study is in progress. Subsequent studies suggest further exploration of the underlying mechanism of AB4 in ERS and the deep relationship between ERS and apoptosis.

In conclusion, the present study demonstrates that AB4 may mitigate CIRI by inhibiting ERS-mediated apoptosis. Therefore, AB4 represents a promising foundation for the development of novel therapeutic strategies in ischemic stroke.

Different dosages of AB4 were given to Male Sprague–Dawley (SD) rats by intraperitoneal injection. Rats were subjected to 120 min of MCAO cerebral ischemia plus 24 hr of reperfusion. MCAO/R: Middle cerebral artery occlusion; AB4: Anemoside B4

**Table 1:** Rat primer sequences of GRP78, PERK, ATF6, IRE1α and β-actin

Name	Primer sequence
GRP78	Forward: 5′-TCTGGTTGGCGGATCTACTC-3′
	Reverse: 5′-TCTTTTGTCAGGGGTCGTTC-3′
PERK	Forward: 5′-AAGATGGTACAGTGGACGGC-3′
	Reverse: 5′-CCGTGTTCCTGGTGAAATCT-3′
ATF6	Forward: 5′-ATCACCTGCTATTACCAGCTACCAC-3′
	Reverse: 5′-TGACCTGACAGTCAATCTGCATC-3′
IRE1α	Forward: 5′-GACCGGCAGTTGCAGTACAT-3′
	Reverse: 5′-TGGTCTGATGAAGCAAGGTG-3′
β-Actin	Forward: 5′-TGTCACCAACTGGGACGATA-3′
	Reverse: 5′-GGGGTGTTGAAGGTCTCAAA-3′

(A) Representative TTC-stained brain sections and quantification of infarct volume. (B) Neurological deficit score. Data are presented as mean ± SEM (n=6). ****P<0.0001 vs Sham group; #*P*<0.05, ####*P*<0.0001 vs MCAO/R group. MCAO/R, middle cerebral artery occlusion/reperfusion; AB4, Anemoside B4; AB4-L, Low dosage of AB4 (1.25 mg/kg); AB4-M, Middle dosage of AB4 (2.5 mg/kg); AB4-H, High dosage of AB4 (5 mg/kg).

(A) Representative HE-stained brain sections (n=3). (B) Representative Nissl-stained brain sections (n=3). (C) Relative injury cells in the cortex (n=3). Scale bar, 50 μm. Data are presented as mean ± SEM (n=3),****P<0.0001 vs Sham group; ####*P*<0.0001 vs MCAO/R group. MCAO/R, middle cerebral artery occlusion/reperfusion; AB4, Anemoside B4; AB4-L, Low dosage of AB4 (1.25 mg/kg); AB4-M, Middle dosage of AB4 (2.5 mg/kg); AB4-H, High dosage of AB4 (5 mg/kg).

Western blot analysis results of GRP78 in the cortex of rats after the indicated durations of MCAO/R. Data are presented as mean ± SEM (n=3). **P<0.01, ***P<0.001 and ****P<0.0001 vs Sham group. MCAO/R, middle cerebral artery occlusion/reperfusion; GRP78, glucose-regulated protein 78kD.

(A) The relative protein expression of GRP78. (B) Semi-quantitative analysis of GRP78. (C) Western blotting of p-PERK, PERK, ATF6, p-IRE1α and IRE1α. (D) Semi-quantitative analysis result of p-PERK/PERK. (E) Semi-quantitative analyses of ATF6. (F) Semi-quantitative analysis result of p-IRE1α/IRE1α. Data are presented as mean ± SEM (n=6). ****P<0.0001 vs Sham group; #*P*<0.05, ##*P*<0.01, ###*P*<0.001, and ####*P*<0.0001 vs MCAO/R group. MCAO/R, middle cerebral artery occlusion/reperfusion; AB4, Anemoside B4; AB4-L, low dosage of AB4 (1.25 mg/kg); AB4-M, middle dosage of AB4 (2.5 mg/kg); AB4-H, high dosage of AB4 (5 mg/kg); GRP78, glucose regulated protein 78kD;ATF6, activating transcription factor 6; p-, phosphorylated; IRE1α, inositol requiring enzyme 1α; PERK, Protein kinase RNA-like endoplasmic reticulum kinase.

TUNEL staining of brain tissue slices. Nuclei were counterstained with DAPI. TUNEL and DAPI images were merged. TUNEL-positive frequency was statistically analyzed. Scale bar, 100 μm. Data are presented as mean ± SEM (n=3). ****P<0.0001 vs Sham group; ####*P*<0.0001 vs MCAO/R group. MCAO/R, middle cerebral artery occlusion/reperfusion; AB4, Anemoside B4; AB4-L, low dosage of AB4 (1.25 mg/kg); AB4-M, middle dosage of AB4 (2.5 mg/kg); AB4-H, high dosage of AB4 (5 mg/kg).

## Conclusion

This study demonstrated that AB4 significantly alleviated neurological deficits and mitigated pathological damage in the brain tissue of MCAO/R rats. Additionally, AB4 reduced ERS and the apoptosis induced by ERS. Our findings provide robust experimental evidence supporting the potential of AB4 as a preventive and therapeutic agent for CIRI, suggesting that AB4 may serve as a promising candidate drug for CIRI treatment.

## Declaration

We employed Youdao AI Translation to prepare this manuscript.
